# Partnership Dynamics of LGB People and Heterosexuals: Patterns of First Partnership Formation and First Cohabitation

**DOI:** 10.1007/s10680-024-09697-4

**Published:** 2024-03-19

**Authors:** Jeanette Bohr, Andrea Lengerer

**Affiliations:** GESIS – Leibniz Institute for the Social Sciences, Square B6, 4-5, 68159 Mannheim, Germany

**Keywords:** Life course, Partnership formation, Cohabitation, Sexual orientation, LGB people, Heterosexuals

## Abstract

In this study we examine partnership dynamics among people with different sexual orientations in Germany. More specifically, we explore the process of first partnership formation and first cohabitation among men and women who self-identify as heterosexual, homosexual or bisexual. Given the various discriminations against same-sex lifestyles, and the limited opportunities to meet potential partners, we assume that lesbian, gay and bisexual (LGB) people form partnerships later in life and less frequently than heterosexuals. We further expect that the constantly improving social and legal climate for sexual minorities will lead to a reduction in differences in partnership behaviour by sexual orientation. We use retrospectively reported partnership biographies from the German Socio-Economic Panel, which was supplemented in 2019 with a boost sample of sexual and gender minority households. Using discrete-time event history models, we analyse nearly 15,000 episodes of being single and nearly 20,000 episodes of living without a partner in the household. Around 4.5% of these episodes are from people who self-identify as LGB. The results clearly show that patterns of partnership and coresidential union formation differ by sexual orientation. People with a homosexual orientation—and to a lesser extent people with a bisexual orientation—are less likely to enter into a first partnership and a first cohabitation than people with a heterosexual orientation. Significant changes occur across cohorts: LGB people from younger birth cohorts enter (cohabiting) partnerships much earlier and more frequently than those from older cohorts. Thus, the union formation patterns of LGB and straight people have converged slightly.

## Introduction

The diversity of sexual orientations has become increasingly visible, socially accepted and legally recognised in recent decades. Empirical knowledge about people with a lesbian, gay or bisexual (LGB) orientation has also increased. However, studies based on representative data focus mainly on co-resident same-sex partnerships (e.g. Baumle et al., 2009; Black et al., 2000; Lengerer & Bohr, 2019a; Manning & Payne, 2021). Most of these studies are cross-sectional and provide information on the prevalence and social structure of same-sex partnerships. Only a few are longitudinal and deal with the stability of same-sex partnerships. Little is known about entering into same-sex partnerships and the partnership dynamics of LGB people over the life course. Thus, it remains unclear whether same-sex partnerships are rare because few people identify as LGB, because LGB people are less likely to enter into a partnership, because they move in together less often and/or because they separate more often than people with a heterosexual orientation.

Intimate partner relationships are not only subjectively meaningful but also relevant for society. They have an impact on personal well-being as well as on physical and mental health (Chen & van Ours, 2018; Koball et al., 2010; Lamb et al., 2003). They are designed to provide mutual support and constitute an important social resource. Moreover, cohabiting partnerships in particular offer economic advantages and can thus be considered an aspect of social inequality.

Against this background, and in a societal context in which LGB people are still a discriminated minority despite all the progress made in recent years (Gerhards, 2010; Slenders et al., 2014; Steffens & Wagner, 2004), we examine the partnership behaviour of LGB people in Germany and whether it differs from that of heterosexuals.^1^ We adopt a longitudinal perspective and focus on two steps of partnership formation, namely entry into a committed partnership (with a duration of at least 6 months) and entry into a coresidential union. We do not consider marriage, as it was not opened to same-sex couples in Germany until 2017.^2^

We focus on entry into *first* partnership and into *first* coresidential union in the life course. Both events usually take place at a younger age and are influenced by different factors than are transitions into further partnerships and coresidential unions. For example, Rapp (2018) shows that socioeconomic status is insignificant for partnership formation at younger ages, whereas it increases the likelihood of partnership formation in middle adulthood. Moreover, first partnership and first cohabitation are important stages in the life course that are also important for later partnership behaviour (Meier & Allen, 2009; Sassler, 2010).

We are interested here in both the *timing* and the *intensity* of first partnership formation and first cohabitation. Thus, we look at the temporal dynamics of transitions and their frequency, as most people enter into a partnership at some point and move in with a partner sooner or later. However, some people remain permanently single and/or live alone permanently. Given the importance of intimate relationships, the long-term absence of such relationships seems to us to be an important aspect of inequality. Knowledge about differences in this regard between LGB people and heterosexuals can thus contribute to a broader understanding of the economic, social and health-related disparities between these groups that have been demonstrated in many studies (e.g. Kasprowski et al., 2021; Liu & Reczek, 2021; Meyer, 2003; Thomeer & Reczek, 2016).

To our knowledge, the present study is not only the first to examine the partnership behaviour of LGB people in Germany but also the first to examine how it is changing. To this end, we compare the partnership trajectories of cohorts who were born during or after World War II and grew up in a period in which the social and legal recognition of LGB people increased considerably in Germany and many other Western countries (Fernández & Lutter, 2013; Slenders et al., 2014).

Such analyses rely on the availability of suitable data. Although there are already various large-scale surveys in which data on partner relationships are collected on a longitudinal basis, these surveys either do not include information on sexual orientation and/or the gender constellation of respondents’ partnerships, or the number of cases is too small.^3^ The German Socio-Economic Panel (SOEP) boost sample of sexual and gender minority households, which was recruited in 2019, is the first dataset for Germany that allows us to examine the partnership biographies of LGB people on a representative basis (Fischer et al., 2022).

In what follows, we provide a brief overview of previous findings on LGB partnering and cohabitation behaviour and present some theoretical considerations. From these, we derive expectations regarding differences between the partnering processes of LGB people and heterosexuals, and changes among LGB people across cohorts. After describing the data and methods, we report the results of our analyses, beginning with the process of first partnership formation and then focusing on the process of first coresidential union formation. The paper concludes with a brief summary and discussion of our main findings.

## Previous Research

Research for Germany shows that although same-sex cohabitation is quite rare (less than 1% of all cohabiting couples are same-sex), it has increased over time and across birth cohorts (Lengerer & Bohr, 2019a). An increase in same-sex cohabitation can also be observed in other Western countries (e.g. Black et al., 2000; Lofquist et al., 2012; Statistics Canada, 2012). Regarding the role of sexual orientation, studies suggest that—compared with heterosexuals—gays and lesbians are less likely to be partnered, and same-sex couples are less likely to live together (Lengerer & Bohr, 2019a; Strohm et al., 2009). However, as the aforementioned studies are based on cross-sectional data, it remains unclear whether gays and lesbians actually enter into partnerships less often and move in together less often, or whether these differences are due (also) to differing separation risks.

Previous studies on the dynamics of sexual minority partnership processes have focused mainly on partnership stability. Many of these studies conclude that same-sex partnerships are less stable than opposite-sex partnerships (Andersson et al., 2006; Joyner et al., 2017; Kalmijn et al., 2007; Lau, 2012; Wiik et al., 2014). The higher risk of separation among same-sex couples is explained on the one hand by the fact that they are mostly childless and more often live together unmarried (Andersson et al., 2006), and on the other hand by the fact that, as a minority in a heteronormative society, LGB couples are exposed to a higher level of stress (Joyner et al., 2017). However, some studies have found little or no difference in partnership stability between same-sex and different-sex couples (Badgett & Herman, 2013; Manning et al., 2016), and other studies have found differences mainly between lesbian and all other couples (Andersson et al., 2006; Joyner et al., 2017).

To date, there have been few representative studies on whether the processes of entering into partnerships and moving in together differ by sexual orientation. The few studies we are aware of address different aspects: Lin et al. (2019) use retrospectively collected data to examine relationship formation among young adults in Taiwan and show that factors associated with relationship formation, such as gender, relationship experience and educational status, have broadly similar effects on same-sex and opposite-sex partnerships. However, they find that same-sex partnerships are formed somewhat later. Using data from two British birth cohort studies, Strohm (2012) shows that same-sex cohabitation is entered into later than opposite-sex cohabitation. However, his expectation that this was due to the fact that “during the coming-out process, individuals first enter different-sex union(s), delaying same-sex union entry” was not supported by the data (Strohm, 2012, p. 23). Strohm (2012) concludes that “a more likely explanation is that same-sex cohabiters delay union formation until they are older and have achieved independence from family and other third parties” (p. 23). This is in line with the finding of a US study by Rosenfeld and Kim (2005) that same-sex couples are particularly likely to live geographically far away from their families of origin. Prince et al. (2020) examine the process of same-sex couples in the United States moving in together for the first time. Focusing primarily on family and social contextual effects that support entering into a partnership, they show that both coming out to parents and living in an environment with a high concentration of same-sex couples have positive effects on the cohabitation rate of gay and lesbian couples. Also for the United States, Orth and Rosenfeld (2018) conclude on the basis of a representative and longitudinal dataset that the dynamics of entering into romantic relationships and moving in together are quite similar between opposite-sex and same-sex couples, but that gay couples have shorter periods of acquaintance before entering into a romantic relationship. For the United Kingdom, Ophir et al. (2023) show using sequence analysis that both gay men and lesbian women are less likely to belong to “partnership-centered trajectories” than their heterosexual counterparts, and that younger cohorts of gay men and lesbian women are more likely to be partnered than older cohorts. For Germany, we are not aware of any longitudinal study that examines the processes of entering into partnerships and moving in together as a function of people’s sexual orientation.

Little is known about the partnership behaviour of bisexual people. This is due in part to the fact that researchers often focus only on the gender constellation within couples rather than their sexual orientation. Studies on people with a bisexual orientation point in particular to specific experiences of discrimination by both heterosexual and gay/lesbian people (Doan Van et al., 2019; Sarno et al., 2020). Furthermore, negative attitudes towards bisexuality on the part of potential partners can negatively affect the interest in a partnership with bisexual people as well as the stability of such partnerships (Armstrong & Reissing, 2014). Studies on the stability of sexual orientation also show that among the non-heterosexual orientations, bisexuality is the least stable over the life course (Mock & Eibach, 2012; Savin-Williams et al., 2012).

## Theoretical Considerations

Partnerships have a high status in society and are the most common way of living in the adult population. The many reasons for establishing a partnership can be seen as largely independent of sexual orientation. In addition to benefits such as emotional support and physical closeness in partnerships, establishing a joint household also offers economic advantages. In fact, studies show that the desires and expectations of LGB people regarding intimate partnerships and long-term relationships are very similar to those of heterosexuals (Barrios & Lundquist 2012; Frost, 2011; Hank & Wetzel, 2018; Potârcă et al., 2015). Nevertheless, theoretical considerations on the partnership dynamics of LGB people point to some differences from those of heterosexuals, which will be discussed in more detail below.

### Differences in the Partnership Formation Process

One obvious difference between LGB people and heterosexuals lies in the structural opportunities to meet and get to know potential partners. Because the partner market for same-sex partners is not only significantly smaller but also less transparent and harder to access, it is much more difficult for LGB people to connect with potential partners. The search for a partner is also made more difficult by the fact that sexual orientation is not immediately recognisable as a relevant characteristic for partner selection. In addition, bisexually oriented people may face the problem that potential partners may not perceive their bisexual orientation as being clear enough. As a result, dating can be costly and time-consuming for LGB people, thereby leading to delays in partnership formation.

The particular stress factors that LGB people face as members of a stigmatised group can also be seen as barriers to entering into a partnership (Meyer, 2003). These stressors include, for example, experienced and anticipated stigmatisation and discrimination, concealment of one’s sexual orientation, and internalised homophobia (LeBlanc et al., 2015). Several studies have found negative associations between minority stress and the mental and physical health of LGB populations (Cao et al., 2017; Kasprowski et al., 2021; Meyer, 2003). Fears of rejection and internalised negative beliefs can result in LGB people not entering into partnerships in the first place, investing less in relationships, and moving in together less often (Joyner et al., 2017). The risk that their relationship will be rejected by the social environment is particularly high for same-sex couples (Frost & Gola, 2015). A study from Germany confirms that people in same-sex relationships are significantly more likely than people in heterosexual relationships to express concerns that their partner will be rejected by family or friends (Hank & Wetzel, 2018). People with a bisexual orientation are also exposed to unique stressors, as negative attitudes and discrimination can come from both heterosexual and gay/lesbian people, and it is more difficult for bisexuals to find a supportive community (Feinstein & Dyar, 2017; Perales, 2016).

Another crucial difference is that the benefits of long-term relationships such as marriage and parenthood, which are open to heterosexual couples, have long been unavailable to or very limited for same-sex couples. Access to parenthood is particularly limited in gay partnerships, but also in lesbian partnerships (Black et al., 2007). These aspects are discussed as reasons for the lower commitment and stability of same-sex partnerships (Andersson et al., 2006), and they also lead to lower incentives to move in together.

Finally, it should be taken into account that there are already differences between LGB people and heterosexuals in the development of their sexual identity. Although German society is now more open to non-heterosexual ways of living, and the choice of partner is freer and more independent of external influences, individuals are still strongly socialised in a heteronormative way. Whereas for heterosexual people the formation of their sexual identity is in line with social norms, this is not the case for LGB people. The development and consolidation of a sexual identity that deviates from the norm is more difficult and takes longer (Heatherington & Lavner, 2008). The longer process of finding one’s sexual identity is seen as one of the reasons why LGB people enter into a partnership later and less often (Prince et al., 2020; Strohm, 2012).

Based on these aspects, we expect that LGB people enter into partnerships later and generally less frequently than heterosexuals, and that moving in with a partner for the first time also occurs later in the life course and less frequently. Cohabitation can be seen as more challenging for LGB persons than for heterosexuals, as the greater visibility must also be accompanied by a willingness to live the relationship openly despite the risk of social rejection. Same-sex couples also have fewer incentives to move in together, because although they benefit from the economic advantages of a joint household, they do not benefit from the advantages of marriage—or at least they have not done so until recently—and because they are mostly childless.

### Changes in the Partnership Formation Process among LGB People

Besides the differences between LGB and straight people mentioned above, there are a number of developments that are likely to have reduced the impact of sexual orientation on the partnership formation process.

The transparency and accessibility of LGB partner markets has increased over time: the internet has immensely facilitated contact opportunities with potential partners, and significantly expanded the available venues for searching for a partner (Potârcă, 2017; Rosenfeld & Thomas, 2012). The structural disadvantages of dating in rural areas compared with the metropolitan context are also less significant due to the possibilities of online dating. In addition, legal and societal liberalisation has led to progressive de-tabooisation, more comings out, and higher visibility of non-heteronormative lifestyles in public spaces.

In Germany and throughout Europe, increasing tolerance and acceptance towards LGB people has been observed in recent years and decades (de Vries, 2021; European Commission, 2019; Gerhards, 2010). Compared with other (Western) European countries, Germany is still not one of the most liberal societies (Kuntz et al., 2015; Smith, 2011). Although a broad acceptance of same-sex lifestyles can be observed in many social milieus in Germany today (Küpper et al., 2017), LGB persons still face discrimination not only in everyday life but also in the labour market (de Vries et al., 2020; Steffens & Wagner, 2004). Nevertheless, it is likely that due to cultural change, LGB persons in Germany have become more independent of normative restrictions in their partnership behaviour.

The legal situation for LGB people in Germany has also been liberalised in recent decades. One important legislative change was the decriminalisation of consensual sexual acts between adult men in 1994.^4^ Other major steps towards legal equality after German reunification in 1990 were the Civil Partnership Act (LPartG), which entered into force in 2001, and the Act on the Introduction of the Right to Marriage for Persons of the Same Sex, which entered into force in 2017 and granted same-sex couples almost the same rights and legal recognition by the state as heterosexual couples. Although the legal recognition of same-sex partnerships has taken longer in Germany compared with many other (Western European) countries, the benefits of long-term relationships for LGB persons have increased significantly since marriage (and to some extent parenthood) became possible.

We expect that due to the growing social and legal acceptance as well as the increasing visibility of LGB people, the disadvantages they face by entering into partnerships and cohabiting unions will be less pronounced in younger cohorts than in older cohorts. As the developments described above are strongly interlinked, it is hardly possible to determine the relative impact of the individual mechanisms in detail. However, the overall picture suggests that patterns of first partnership formation among LGB people change across cohorts, with first partnership and first cohabitation occurring both earlier in life and more frequently in younger cohorts.

## Data and Methods

Our analyses are based on data from the German Socio-Economic Panel (SOEP), which is the largest household panel survey in Germany (Goebel et al., 2019).^5^ We include all SOEP respondents who were asked a question about sexual orientation. This question was asked for the first time in 2016 and again in 2019 when the SOEP was supplemented with a boost sample of sexual and gender minority (SGM) people (Sample Q) comprising 477 randomly selected households with individuals who self-identify as lesbian, gay, bisexual or with another non-heterosexual orientation. The introduction of Sample Q brought the total number of SGM households in the SOEP to 822 (de Vries et al., 2021).

Recruitment of households for Sample Q was done in two phases (Fischer et al., 2022): In the first phase, a random telephone screening of the general adult population in Germany was conducted, where roughly 75,000 people were interviewed and asked about their sexual orientation and gender identity. All those who chose an answer other than “heterosexual” were screened as part of the target group and were invited to take part in the SOEP survey in the second phase. Around 30% of the invitees from the target group actually took part.

Data on first partnership and first cohabitation in the life course were taken from respondents’ partnership biographies, which were collected retrospectively. These biographies include all partnerships that lasted at least 6 months or are still ongoing.^6^ The starting date of each partnership episode was recorded in years, and a maximum of three previous partnerships could be reported in addition to the current one.^7^

### Dependent Variables

Our analysis focuses initially on entry into first partnership. For this purpose, we estimate an event history model in which the transition rate into first partnership is the dependent variable. This rate is calculated on the basis of the time in years that a person spends without a partner from the age of 12, which is the earliest age at which intimate relationships develop. The process ends with the beginning of the first relationship (14,654 episodes) or censoring (305 episodes). If the starting date of the first relationship is not reported, we exclude the person from the analysis. Also excluded from the analysis are respondents who were not born in Germany and who migrated after the age of 12, as their partnership biographies started under different conditions.

We then analyse the process leading to first cohabitation. Here, the dependent variable in the event history analysis is the rate of transition into first cohabitation (irrespective of whether cohabitation is accompanied by marriage). The analysis begins at the age of 16—the age at which a “risk” of cohabitation begins. The timescale ends with the beginning of a first coresidential relationship (15,341 episodes) or censoring (4322 episodes). Respondents with incomplete information on moving in together are not included.^8^ As in the first part of the analysis, this applies also to respondents who were born abroad and migrated to Germany after the age of 12.

### Independent Variables

Our main independent variable is sexual orientation. The SOEP asks about self-identification as heterosexual (attracted to the other gender), homosexual (gay/lesbian, attracted to the same gender) or bisexual (attracted to both genders). In addition, respondents interviewed in 2016 could indicate “none of these”, and Sample Q respondents interviewed in 2019 could indicate “other orientation”. In both cases, respondents had the option to select “no answer”. Those who did not (clearly) assign themselves are combined and remain as a separate group in our analyses. This group comprises around 4% of all respondents.

The data on sexual orientation refer to the time of the survey, not the time of entering into a partnership. Therefore, we must treat sexual orientation as time-invariant, although recent studies have shown that it can vary over the life course (Kinnish et al., 2005; Mock & Eibach, 2012; Savin-Williams et al., 2012). Moreover, sexual orientation does not always correspond to the gender constellation within the partnership (Dewaele et al., 2014, pp. 264–266.; Kühne et al., 2019). As the SOEP does not contain information on the gender of previous partners, we consider only sexual orientation, not gender homogamy or heterogamy.

The other independent variables are birth cohort, gender and education. The birth cohorts are grouped into four categories: cohorts born in 1940–1955, 1956–1970, 1971–1985, and 1986–2001. The first group comprises the war and post-war cohorts who grew up in a traditional environment that was marked by the legal prohibition of homosexuality. Those born between 1956 and 1970 were socialised in a more liberal era, characterised in West Germany by educational expansion and the rise of women’s and student movements. Those born between 1971 and 1985 grew up in a society in which homosexual lifestyles became increasingly visible and socially accepted. The youngest cohort groups observed here—those born from 1986 onwards—benefited most from the legal recognition of same-sex partnerships. They were aged 15 years or younger when the German Civil Partnership Act (LPartG) entered into force in 2001, and they were aged between 16 and 31 years when marriage was opened to same-sex couples in 2017. We exclude those born before 1940 from our analyses.

Gender is coded dichotomously (male, female), as it is measured in that way in the SOEP Core samples.^9^ Education is used as a time-dependent variable, indicating whether a person (a) is still in education; (b) has neither a higher education entrance qualification nor a vocational training qualification (low education); (c) has a higher education entrance qualification or a vocational training qualification (medium education); or (d) has completed higher education (high education).

Control variables are size of place of residence at age 15 (city vs. other); growing up in the GDR (only for respondents born before 1980; measured by living in the GDR in 1989, the year of the fall of the Berlin Wall); and social background as measured by the respondent’s father’s education (low, medium or high, as in the case of respondent’s own education).

A description of the samples is provided in Appendix Tables 3 and 4. Appendix Table 3 shows the distribution of characteristics among respondents whose transition into first partnership is analysed; Appendix Table 4 shows the distribution of characteristics among respondents whose transition into first cohabitation is analysed. As LGB people account for 4.5% of all respondents in both samples, they are overrepresented compared with the general population (where the percentage is estimated to be between 1 and 3%, depending on the study, e.g. Kroh et al., 2017, p. 338). Nevertheless, their absolute number is low, which limits the statistical significance of our results. There is little gender difference in the proportion of LGB people in our samples. However, whereas about 3% of male respondents self-identify as homosexual, and about 1.5% self-identify as bisexual, the proportions are exactly the other way round among female respondents. This is consistent with findings from other countries (e.g. Gates, 2011; Hayes et al., 2012; Perales, 2016). Similarly, our samples reveal the well-known educational distribution among lesbians and gays compared with heterosexuals: higher educational attainment is more common among lesbians and gays, which is due only partly to the fact that they belong to younger cohorts (Andersson et al., 2006, pp. 88–89; Aspinall, 2009, pp. 95–98; Black et al., 2000; Lengerer & Bohr, 2019b; Lengerer & Schroedter, 2022, pp. 169–170).

### Methods

The partnership dynamics of LGB people and heterosexuals are studied using event history analysis. Because time is measured in years, we estimate a discrete-time logit model (Allison, 1982; Yamaguchi, 1991) where the dependent variable is the log odds of first (cohabiting) partnership formation. The model can be formalised as:

$$\ln \left( {\frac{P\left( t \right)}{{1 - P\left( t \right)}}} \right) = \beta_{0} + \beta_{1} t + \beta_{2} {\text{ln}}\left( t \right) \, + \beta_{i} x_{i} + \beta_{j} x_{jt}$$

where *P*(*t*) is the conditional probability of entering a first (cohabiting) partnership for a person at year *t* since age 12 (age 16), given that the person has not yet had a (cohabiting) partner or been censored prior to year *t*. The set of time-constant covariates is represented by *x*_*i*_, the time-varying covariate education is represented by *x*_*jt*_, and *β*_0_ to *β*_*j*_ are the parameters to be estimated.

For the time effect, we assume that it is non-monotonic: the transition rate into partnership and into cohabitation increases rapidly at the beginning of the process, reaches a maximum soon afterwards and then decreases gradually. In the lower age range, a large number of people start an intimate relationship and often move in together. As people get older, more and more potential partners are already “taken” (or have little interest in a relationship), which slows down the process. Later on, the transition rate converges to zero because some people never enter into a partnership and/or never move in together. In order to model this sickle-shaped transition rate, time *t* is included as a linear and logarithmic term. This specification is flexible and has been used to analyse various processes of partnership and family formation and dissolution in Germany (Klein & Eckhard, 2007; Rapp & Gruhler, 2018; Schmid, 2022).

First, we estimate a model with sexual orientation as the central independent variable. In a second model, by including interaction effects we take account of the fact that the effect of sexual orientation may vary with process time. We then run separate models for individuals with a heterosexual, homosexual or bisexual orientation and determine the effects of all other independent variables within these groups.

To facilitate the interpretation of interaction effects, we graphically present the transition rates predicted from the respective models. We show the cohort effect within the sexual minority groups using estimated survival curves. They indicate the predicted probabilities of having entered into a first partnership or cohabitation by a certain time and are therefore very illustrative.

## Results

### Descriptive Results

**Fig. 1:**
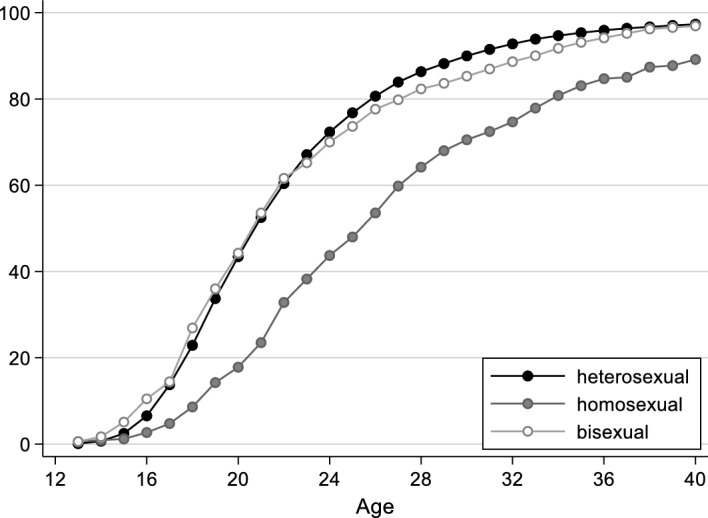
Proportion of people who entered into a partnership, by sexual orientation (life-table estimates, in %). Data source: German Socio-Economic Panel, survey years 2016 and 2019

Figure 1 presents life-table estimates of first partnership formation for self-identified heterosexuals, homosexuals and bisexuals. The x-axis shows age in years; the y-axis shows the cumulative proportion of individuals who had entered into a partnership. There are clear differences by sexual orientation. Among gays and lesbians, the proportion who have entered into a partnership is considerably lower than among heterosexuals at any age. By age 20, for example, only 18% of all gays and lesbians have entered into a partnership, whereas 43% of all heterosexuals have already done so. Although this difference becomes smaller in later stages of the life course, it persists, so that gays and lesbians not only enter into partnerships considerably later than heterosexuals, but ultimately also less frequently. At age 40, 11% of all lesbians and gays are still single, meaning that they have never had a partnership, which is the absolute exception among heterosexuals at that age (< 3%). The latter is also true of people with a bisexual orientation, who hardly differ from straight people in this regard.

**Fig. 2:**
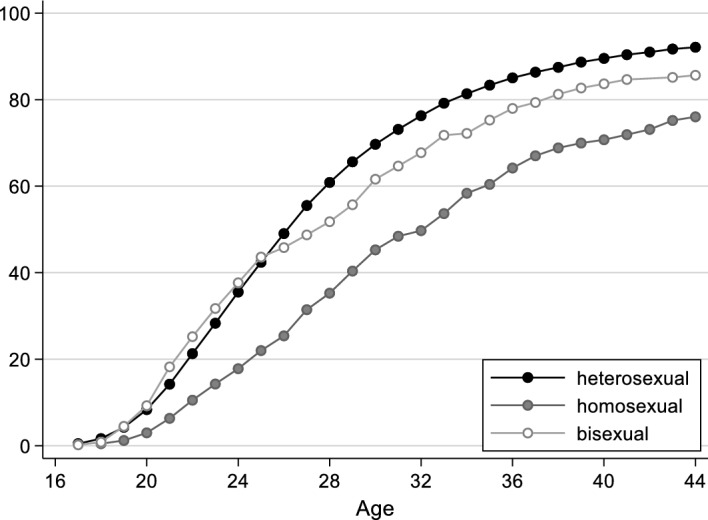
Proportion of people who entered into a coresidential union, by sexual orientation (life-table estimates, in %). Data source: German Socio-Economic Panel, survey years 2016 and 2019

In Fig. 2 we show the results for the transition to first cohabitation. Compared with entering into the first partnership, moving in with a partner for the first time takes place less often overall, which is due to the fact that not every partnership leads to a cohabitation. As with the results for first partnership, there are also clear differences by sexual orientation here. The proportion of homosexually oriented people who moved in with a partner for the first time is lower over the entire life course than among heterosexually oriented people. Among heterosexuals, half have already moved in with a partner for the first time by the age of 26; among gays and lesbians, this is not the case until the age of 32. Among those who self-identify as bisexual, the proportion of people who have already moved in with a partner once is also lower than among heterosexuals, but the differences are much smaller, and almost non-existent until the mid-twenties.

The speed and intensity of the process differ by sexual orientation: among heterosexually oriented individuals, first cohabitations occur mainly between the ages of 20 and 30 and become progressively less frequent thereafter, whereas the increase among homosexually oriented individuals is more consistent across age. At the end of the age ranges shown here (44 years), around 93% of all heterosexual respondents have moved in with a partner, as compared with 87% of bisexual respondents and only 78% of lesbian and gay respondents. In other words, lesbians and gays in particular have a significantly higher probability of never moving in with a partner, or of doing so only at a later age.

### Multivariate Results

To analyse the partnership dynamics of LGB people and heterosexuals, we estimate logit models. Table 1 contains logit models predicting first partnership formation, and Table 2 contains logit models predicting first coresidential union formation. The reported beta coefficients can be interpreted as the log odds of having a transition relative to not having a transition for each person at year *t*. Positive values indicate an increased likelihood of starting a first (cohabiting) relationship, negative values indicate a reduced likelihood, and values close to zero indicate no effect.

**Table 1:** Discrete-time logit models predicting first partnership formation (β coefficients)

	Model 1	Model 2	Model 3	Model 4	Model 5
	All	All + IA	Heterosexuals	Homosexuals	Bisexuals
*t*	− 0.144***	− 0.149***	− 0.149***	− 0.108***	− 0.093***
ln(*t*)	2.109***	2.144***	2.146***	2.297***	1.603***
Sexual orientation (ref. heterosexual)					
Homosexual	− 0.725***	− 1.451***			
Bisexual	− 0.297***	0.407			
Other, no answer	− 0.220***	− 0.206			
Process time × Sexual orientation					
* t* × homosexual		0.028			
* t* × bisexual		0.046*			
* t* × other, no answer		0.025			
ln(*t*) × homosexual		0.155			
ln(*t*) × bisexual		− 0.552**			
ln(*t*) × other, no answer		− 0.127			
Birth cohorts (ref. 1956–1970)					
1940–1955	0.090***	0.088***	0.087**	− 0.595*	− 0.015
1971–1985	0.351***	0.350***	0.338***	0.406**	0.727***
1986–2001	0.894***	0.896***	0.879***	1.272***	1.020***
Gender (ref. male)					
Female	0.476***	0.477***	0.481***	0.285*	0.264^+^
Education (ref. low)					
Medium	0.065	0.061	0.087*	0.119	0.000
High	0.096^+^	0.095^+^	0.108*	0.393	0.048
Still in education	0.036	0.031	0.061	0.110	0.106
No answer	− 0.282**	− 0.289**	− 0.196^+^	0.178	1.602
Place of residence, age 15 (ref. other^a^)					
City	− 0.094***	− 0.093***	0.098***	0.231	− 0.033
Socialised in the GDR (ref. no, other)					
Yes	0.001	0.003	− 0.001	0.168	− 0.136
Father’s education (ref. low)					
Medium	− 0.002	− 0.003	0.004	− 0.805**	− 0.052
High	− 0.102**	− 0.103**	− 0.094*	− 1.129***	− 0.015
Other^a^	− 0.101*	− 0.102*	− 0.100*	− 0.733*	0.035
Constant	− 5.425***	− 5.445***	− 5.475***	− 6.416***	− 5.190***
Log likelihood	− 43,706	− 43,689	− 39,862	− 1,040	− 1,011
*N* (person-years)	148,879	148,879	133,979	4,971	3,503

Data source: German Socio-Economic Panel, survey years 2016 and 2019

*IA* Interaction, *GDR* German Democratic Republic

^+^*p* < 0.10; * *p* < 0.05; ** *p* < 0.01; *** *p* < 0.001.

^a^Including no answer, not asked, not applicable, don’t know (only father’s education)

**Table 2:** Discrete-time logit models predicting first coresidential union formation (β coefficients)

	Model 1	Model 2	Model 3	Model 4	Model 5
	All	All + IA	Heterosexuals	Homosexuals	Bisexuals
*t*	− 0.101***	− 0.102***	− 0.102***	− 0.070***	− 0.104***
ln(*t*)	1.216***	1.233***	1.233***	1.062***	1.181***
Sexual orientation (ref. heterosexual)					
Homosexual	− 0.612***	− 0.783*			
Bisexual	− 0.247***	0.262			
Other, no answer	− 0.246***	− 0.005			
Process time × Sexual orientation					
* t* × homosexual		0.014			
* t* × bisexual		− 0.008			
* t* × other, no answer		0.018			
ln(*t*) × homosexual		− 0.006			
ln(*t*) × bisexual		− 0.208			
ln(*t*) × other, no answer		− 0.204			
Birth cohorts (ref. 1956–1970)					
1940–1955	0.175***	0.174***	0.170***	− 0.641**	0.220
1971–1985	0.215***	0.214***	0.211***	0.101	0.608***
1986–2001	0.173***	0.170***	0.181***	0.431*	0.323^+^
Gender (ref. male)					
Female	0.364***	0.364***	0.358***	0.278*	0.256^+^
Education (ref. low)					
Medium	0.030	0.030	0.039	− 0.293	0.081
High	0.063	0.061	0.060	− 0.141	0.538^+^
Still in education	− 0.426***	− 0.426***	− 0.421***	− 0.878**	0.203
No answer	− 0.211**	− 0.211**	− 0.186*	0.255	1.231*
Place of residence, age 15 (ref. other^a^)					
City	− 0.051*	− 0.051*	− 0.056**	0.073	0.012
Socialised in the GDR (ref. no, other)					
Yes	0.046*	0.046*	0.051*	0.222	0.023
Father’s education (ref. low)					
Medium	− 0.023	− 0.023	− 0.008	− 0.370	− 0.189
High	− 0.091*	− 0.091*	− 0.076*	− 0.410	− 0.147
Other^a^	− 0.212***	− 0.212***	− 0.207***	− 0.550	− 0.522
Constant	− 4.005***	− 4.025***	− 4.042***	− 3.965***	− 4.481***
Log likelihood	− 51,853	− 51,844	− 47,521	− 1,060	− 1,037
*N* (person-years)	207,916	207,916	187,079	6,264	4,732

Data source: German Socio-Economic Panel, survey years 2016 and 2019

*IA* Interaction, *GDR* German Democratic Republic

^+^ *p* < 0.10, * *p* < 0.05, ** *p* < 0.01, *** *p* < 0.001.

^a^Including no answer, not asked, not applicable, don’t know (only father’s education)

The effects of *t* and ln(*t*) indicate the temporal pattern of transitioning. According to these values, the probability of transitioning into a partnership and into cohabitation increases steeply in all models at first, reaches a maximum, and then gradually decreases again. The temporal shape of the logit function is thus clearly non-monotonic and is appropriately modelled by *t* and ln(*t*).

The models of transition to first partnership are presented in Table 1. Model 1 includes all respondents and shows clear and statistically significant differences by sexual orientation. People who self-identify as gay or lesbian have a relatively low propensity to enter into a first partnership. The log odds of first partnership formation at time *t* are 0.725 times lower for gays and lesbians than for heterosexuals. People with a bisexual orientation also have a lower transition rate than people with a heterosexual orientation, but the coefficient is smaller compared with people with a homosexual orientation. Thus, the descriptive results are confirmed with respect to gays and lesbians. However, whereas the descriptive results indicate that patterns of first partnership formation hardly differ between bisexuals and heterosexuals (Fig. 1), the multivariate model shows a statistically significant difference.

**Fig. 3:**
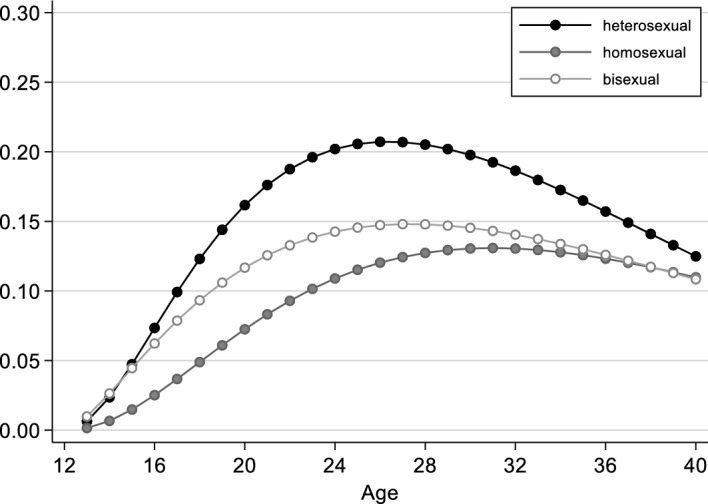
Women’s rates of transition into first partnership, by sexual orientation (from Model 2, Table 1; all other independent variables are set to the modal category: born 1956–1970, medium educated, not resident in a city at age 15, not socialised in the GDR, father medium educated)

The effect of sexual orientation is modelled as a proportional effect in Model 1, meaning that it has the same shape in all groups. To allow for different transition rates, we extend Model 2 (Table 1) to include an interaction effect between process time and sexual orientation. Figure 3 shows (for women) that the propensity to form a first partnership rises less steeply for those with a homosexual orientation than for those with a heterosexual or bisexual orientation, that it reaches its maximum later, and then decreases more slowly. The differences in partnership formation by sexual orientation are thus more pronounced in early stages of the life course than in later stages, where the differences between gays/lesbians and heterosexuals become smaller, and those between gays/lesbians and bisexuals even disappear. However, in later stages, there are also only a few individuals “at risk”, who generally show a low propensity to form partnerships.

We now estimate separate models for people with different sexual orientations (Models 3–5, Table 1). The model for heterosexuals (Model 3) confirms earlier findings (e.g. Kolk, 2015; Konietzka & Tatjes, 2014; Manning et al., 2014): in the comparison between cohorts, the transition rate first decreases slightly (it is lowest in the reference cohort group born 1956–1970) and then increases significantly, showing that first couple relationships are formed earlier in the younger cohorts. Women have higher log odds of partnership formation than men, as they are on average younger when they form partnerships, and they choose older men as partners. The coefficients for all other variables are weak and/or not statistically significant, including the coefficient of own education. The latter finding is not surprising, as first partnerships are often established during education and involve relatively low commitment.

**Fig. 4:**
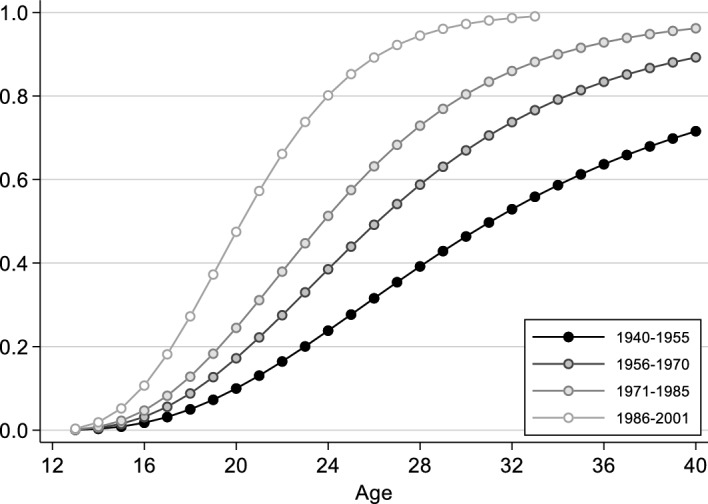
Predicted cumulative probability of first partnership formation among lesbian women, by cohort (from Model 4, Table 1; all other independent variables are set to the modal category: medium educated, not resident in a city at age 15, not socialised in the GDR, father medium educated)

We find clear cohort differences among people with a homosexual orientation (Model 4, Table 1): in all cohort groups, the likelihood of partnership formation is significantly higher than in the respective preceding cohort group. The extent of the differences can be seen in Fig. 4 (for lesbian women), which shows the cumulative probability of partnership formation for different cohorts, predicted from Model 4. It can be clearly seen that the process of partnership formation in the younger cohorts of lesbian women is much faster than in the older cohorts, and that the intensity of partnership formation—that is, the maximum level reached—increases continuously. As expected, people with a homosexual orientation from the younger cohorts enter into partnerships earlier and more frequently than people with a homosexual orientation from the older cohorts, and thus come closer to straight people in this regard.

Homosexually oriented people also differ by gender. Lesbian women have 0.285 higher log odds of partnership formation than gay men. This may be due to the fact that regardless of their sexual orientation, women generally develop an interest in partnership and sexuality earlier in the life course than men do. It is also possible that gay men need more time to form their sexual identity than lesbian women do, and that it is also more difficult for them to live their identity in the face of greater discrimination.^10^ The effect of own education is not significant, but—as education can be seen as an indicator for liberal attitudes—it points in a plausible direction: a higher level of education increases the transition rate into partnership for gays and lesbians.

In the model for bisexuals (Model 5, Table 1), there are also significant differences between cohorts, which are similar to those for gays and lesbians. People with a bisexual orientation are significantly more likely to enter into a first partnership in the younger cohorts than in the older cohorts born between 1956 and 1970. However, the differences are rather moderate, and none are present between the oldest two cohort groups (1940–1955 and 1956–1970). Differences according to gender (higher rates of first partnership formation among bisexual women) can also be observed.

The logit models predicting the transition to first coresidential union are displayed in Table 2. Model 1 indicates that the differences by sexual orientation persist when controlling for other variables in the model: compared with respondents with a heterosexual orientation, the log odds of moving in with a partner are significantly lower for respondents with a bisexual orientation, and even more so for respondents with a homosexual orientation. This is in line with our expectation that especially people with a homosexual orientation experience their first cohabiting relationship with a partner later in life, and that they do so less often than heterosexuals.

**Fig. 5:**
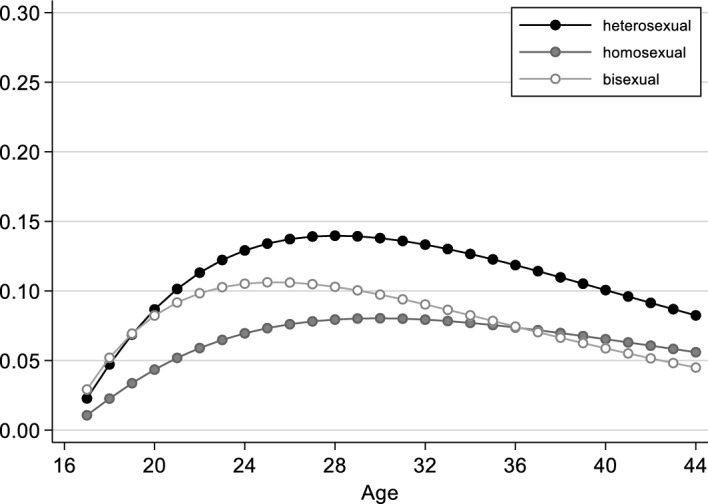
Women’s rates of transition to first cohabitation, by sexual orientation (from Model 2, Table 2; all other independent variables set to the modal category: born 1956–1970, medium educated, not resident in a city at age 15, not socialised in the GDR, father medium educated)

Figure 5 illustrates women’s rates of transition into first cohabitation by sexual orientation. The figure is based on Model 2 in Table 2, which additionally takes into account the interaction between process time and sexual orientation. As in the case of first partnerships, the transition rates differ by sexual orientation: the tendency to enter into a cohabitation for the first time is not only lower among lesbian women than among heterosexual and bisexual women but also more evenly distributed across the age range.

In the models calculated separately by sexual orientation (Models 3–5, Table 2), it can be seen for heterosexual people (Model 3) that—with the exception of the birth cohort group 1956–1970, which has the lowest transition probability— there are no relevant differences between the cohort groups regarding the timing of first cohabitation. This is in line with results of studies in the United States showing that the age at (first) cohabitation has hardly changed across birth cohorts (Manning et al., 2014; Prince et al., 2020). Looking at gender, we find that women are significantly more likely to enter into cohabitation than men. This result is also plausible, as women in heterosexual relationships are usually younger than their partners (e.g. Kolk, 2015). While the fact that someone is still in education reduces the probability of a transition to cohabitation, the level of education attained does not seem to play a role.

**Fig. 6:**
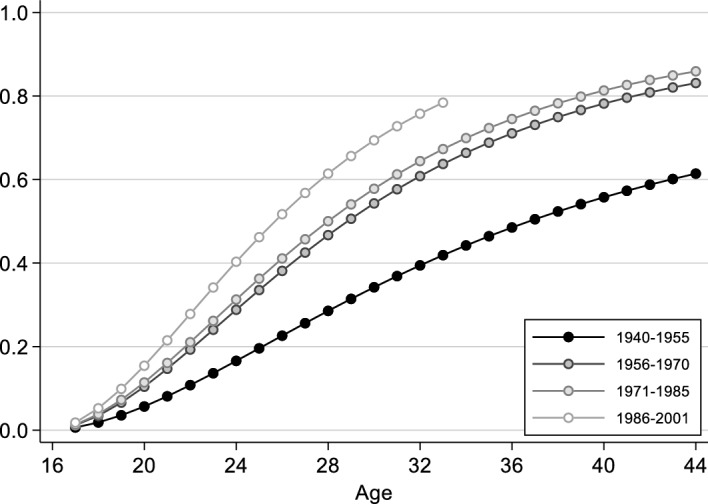
Predicted cumulative probability of first coresidential union formation among lesbian women, by cohort (from Model 4, Table 2; all other independent variables are set to the modal category: medium educated, not resident in a city at age 15, not socialised in the GDR, father medium educated)

We turn now to the results for people with a homosexual orientation (Model 4, Table 2). As in the case of first partnership, the predicted cumulative probability of first cohabitation is shown graphically for the different cohort groups (Fig. 6, for lesbian women). It can be seen that first cohabitation takes place earlier in the younger cohorts of lesbian women, and there are more first cohabitations overall. This indicates that it is easier for younger cohorts of lesbian women to move in with a partner, and that there is a gradual convergence with the partnership trajectories of heterosexually oriented people.

It is not clear why there is a gender difference in the transition to cohabitation among people with a homosexual orientation (Model 4, Table 2).^11^ Similar to the analyses on entry into first partnership, one possible explanation is that lesbian women are less exposed to direct discrimination than are gay men, and that they therefore enter into a committed partnership earlier, are more willing to live it openly, and also find a flat together more easily than two men do. As long as gays and lesbians are still in education, they are significantly less likely to move in together. The level of education, on the other hand, does not appear to be a relevant factor for the timing of first cohabitation.

Cohort differences are also found in the model for bisexually oriented people (Model 5, Table 2): the birth cohort groups 1971–1985 and 1986–2001 enter into a cohabiting relationship earlier and more often than the previous cohort group (1956–1970). All other variables examined show no or only marginally statistically significant results—for example that women have higher log odds of entering into a cohabitation than men do.

To further validate our results on cohort differences, we also explore alternative modelling. We estimate cohort effects among the different sexual orientation groups not only in separate models (Models 3–5, Tables 1–2) but also in the overall model (Model 2, Tables 1–2), where we include interactions between sexual orientation and cohort. Although only some of the interaction effects are statistically significant, the results are consistent with each other and confirm that there is some convergence between heterosexual and LGB people across cohorts in both the transition to first partnership (Appendix Table 5, Model 2a, and Appendix Fig. 7a, b) and to first cohabitation (Appendix Table 6, Model 2a, and Appendix Fig. 9a, b).

## Discussion and Conclusion

In this study we examined the patterns of partnership and coresidential union formation for LGB people and heterosexuals. Taking a longitudinal perspective, we considered both the transition to first partnership and to first cohabitation with a partner over the life course. Our results show that the process of partnership formation in Germany differs significantly by sexual orientation: people with a homosexual orientation enter into a partnership much later and less frequently and move in with a partner considerably later and less frequently than people with a heterosexual orientation. To a lesser extent, this applies also to people with a bisexual orientation, who are thus positioned between lesbians and gays on the one hand and heterosexuals on the other.

The reasons for these differences are probably complex and cannot be determined in detail based on the available data. The assumption that LGB people are less interested in romantic relationships than straight people does not seem very plausible and is not supported by empirical studies. The differences are therefore more likely to be related to the social and structural conditions that LGB people face. The structurally limited possibilities on the partner market for LGB persons are one important condition. Another decisive factor is that the social environment in Germany is heteronormative, and other sexual preferences and lifestyles are perceived as deviant. The negative consequences for non-heterosexual people are multiple, as they have to develop their identity against the prevailing norms, they experience various forms of discrimination, and they have also been legally disadvantaged for a long time. The processes of partnership formation and consolidation are thus made more difficult, take longer and are more prone to disruption.

Another key finding of our study is that the partnership behaviour of LGB people in Germany is changing. As expected, the likelihood of partnership formation and cohabitation among LGB people increased significantly across cohorts, reducing the differences compared with heterosexuals. This also points to the importance of social conditions for individual behaviour. Whereas the subjective value of partnership has presumably remained stable, younger cohorts benefit from society's liberal approach to sexual and gender diversity, the improved legal situation, and easier ways to meet potential partners. As a result, not only do sexual minority identities develop at a younger age, as recent studies show (e.g. Bishop et al., 2020), but these identities can also be lived earlier and more frequently in couple relationships. Despite the positive development, however, our study shows that the patterns of first partnership formation in Germany still differ according to sexual orientation. As partnership living arrangements are relevant to various dimensions of social inequality, inequality persists here. Whether this inequality will continue to decrease and gradually dissolve remains to be seen.

Our study extends previous research on the partnering behaviour of sexual minorities, which is mainly cross-sectional and often limited to individuals in cohabiting same-sex relationships (e.g. Baumle et al., 2009; Black et al., 2000; Lengerer & Bohr, 2019a; Lofquist et al., 2012; Manning & Payne, 2021). A key finding of that research is that same-sex cohabitation is very rare but is increasing over time. Whether sexual minorities are less likely to enter into a (cohabitating) partnership than heterosexuals, and whether younger cohorts of sexual minorities are more likely to enter into a (cohabitating) partnership than older cohorts, cannot be determined based on these studies. The results may also be due to the fact that LGB partnerships are less stable than heterosexual partnerships, that LGB partnerships become more stable over time and/or that more people identify as non-heterosexual. Using a longitudinal approach, we have now been able to show that LGB individuals do indeed differ from heterosexuals in terms of entry into first (cohabiting) partnerships, and that they enter into first (cohabiting) partnerships more frequently across cohorts. Thus, our results indicate a convergence between LGB and heterosexual individuals.

We focused on entry into first partnership and first cohabitation and did not examine subsequent partnerships. Future studies should therefore look at the entire partnership life course of LGB people (Umberson et al., 2015). Based on our findings, it can be assumed that LGB people not only enter into the first but also subsequent partnerships later and less frequently than heterosexuals do. Combined with the lower stability of same-sex partnerships, this would imply that LGB people live alone at different stages of their lives and are therefore more likely to experience loneliness and a greater lack of social support. Using sequence analysis, a paper by Ophir et al. (2023) confirms that living without a partner is common in the life course of LGB people.

With the boost sample of sexual and gender minority people (Sample Q), the SOEP has set an important milestone. However, there are some limitations that affect our analyses. First, although the SOEP includes a comparatively large number of LGB respondents, their number is small in absolute terms, which limits the statistical power of our models. Second, we used retrospectively collected data on partnership biographies, but know the sexual orientation only at the time of the survey. Especially in the older cohorts, we probably classified some people as LGB who self-identified as such only at later stages in their lives. Third, the SOEP does not provide information on the gender of previous partners, so that we could not distinguish between opposite-sex and same-sex partnerships when measuring entry into partnerships. However, the fact that some gays or lesbians may initially identify as heterosexual and may first have heterosexual relationships before entering into homosexual relationships only leads us to underestimate rather than overestimate the differences by sexual orientation.

In the future, more data will be needed that include sufficient numbers of respondents from sexual minorities. These data should be collected longitudinally and contain both prospectively collected information on the respondents’ sexual orientation and their partners’ gender. Although LGB people are a small minority, further empirical analysis of their partnerships and family relationships is worthwhile. Such analyses are not only of interest in their own right but can also contribute to a better understanding of the meaning of gender and gender-specific mechanisms in relationships in general, and—as Evertsson et al. (2021) show—they can stimulate new perspectives on family sociological theories.

## Figures and Tables

**Fig. 7:**
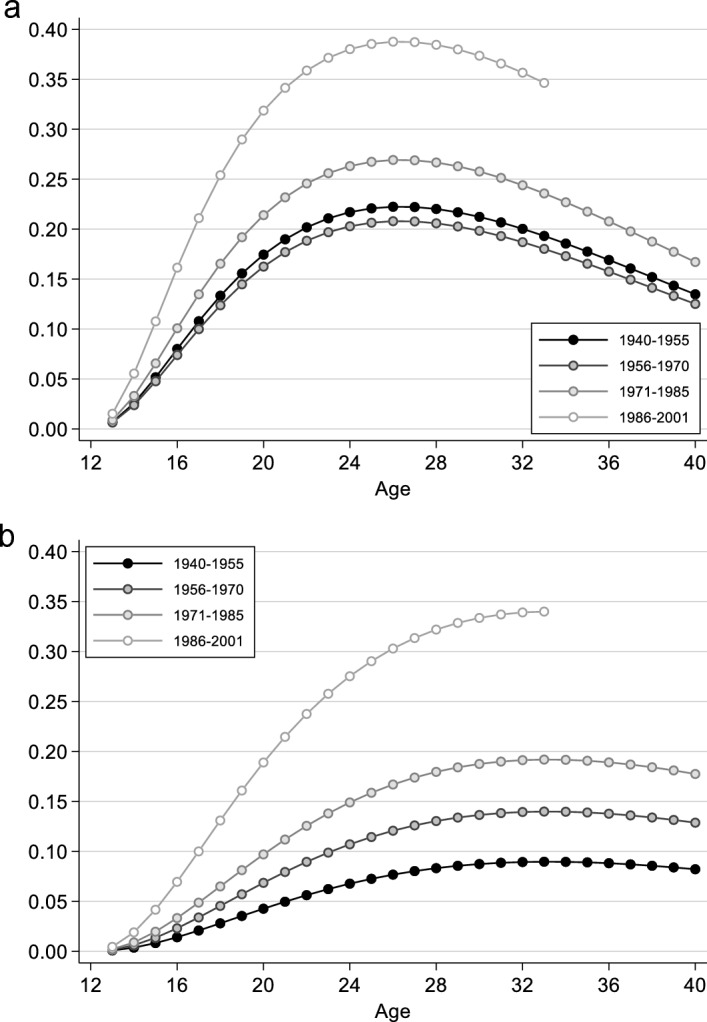
**a** Heterosexual women’s rates of transition into first partnership, by cohort (from Model 2a, Table 5; all other independent variables are set to the modal category: medium educated, not resident in a city at age 15, not socialised in the GDR, father medium educated). **b** Lesbian women’s rates of transition into first partnership, by cohort (from Model 2a, Table 5; all other independent variables are set to the modal category: medium educated, not resident in a city at age 15, not socialised in the GDR, father medium educated)

**Fig. 8:**
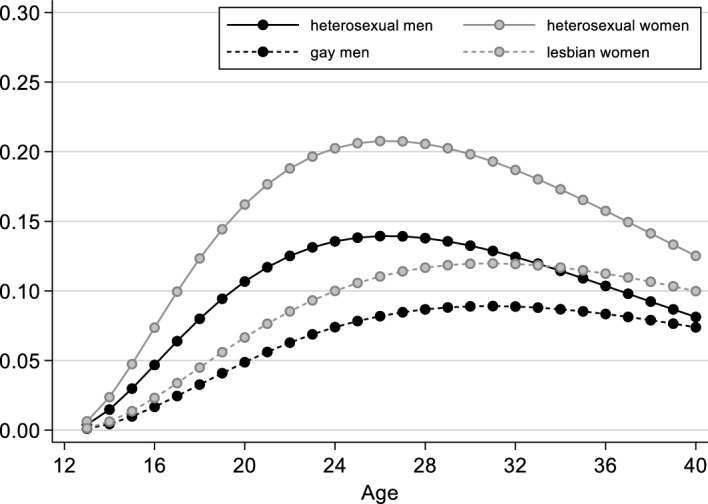
Rates of transition into first partnership, by sexual orientation and gender (from Model 2b, Table 5; all other independent variables are set to the modal category: born 1956–1970, medium educated, not resident in a city at age 15, not socialised in the GDR, father medium educated)

**Fig. 9:**
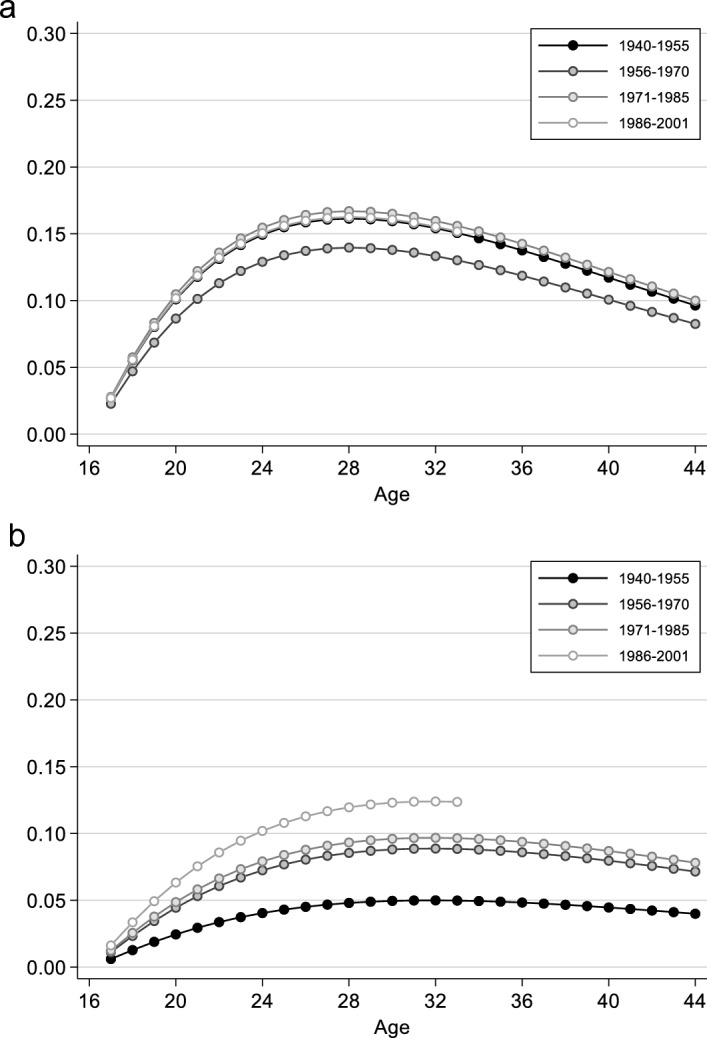
**a** Heterosexual women’s rates of transition into first cohabitation, by cohort (from Model 2a, Table 6; all other independent variables are set to the modal category: medium educated, not resident in a city at age 15, not socialised in the GDR, father medium educated). **b** Lesbian women’s rates of transition into first cohabitation, by cohort (from Model 2a, Table 6; all other independent variables are set to the modal category: medium educated, not resident in a city at age 15, not socialised in the GDR, father medium educated)

**Fig. 10:**
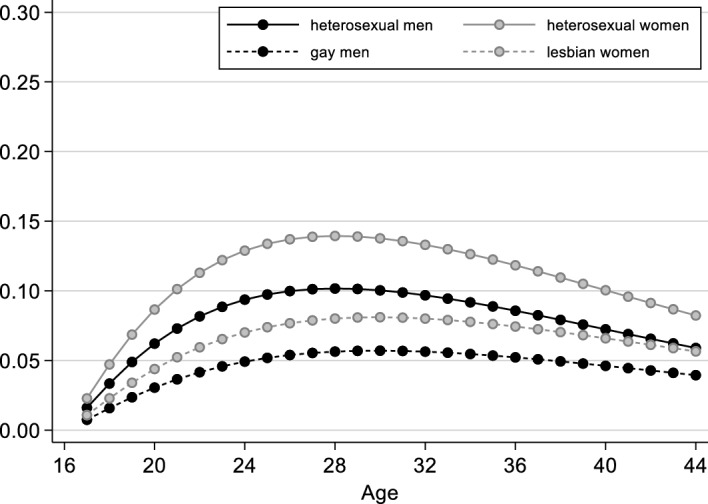
Rates of transition into first cohabitation, by sexual orientation and gender (from Model 2b, Table 6; all other independent variables are set to the modal category: born 1956–1970, medium educated, not resident in a city at age 15, not socialised in the GDR, father medium educated)

## Data Availability

The dataset used in the current study (SOEP-Core, v36, EU Edition, DOI 10.5684/soep.core.v36eu) is available at the Research Data Center of the Socio-Economic Panel (https://www.diw.de/en/diw_01.c.678568.en/research_data_center_soep.html).

## Appendix

See Tables

**Table 3:** Description of the sample used to analyse the process of first partnership formation

	Sexual orientation								All	
	Heterosexual		Homosexual		Bisexual		Other, no answer			
	*N*	Col. %	*N*	Col. %	*N*	Col. %	*N*	Col. %	*N*	Col. %
Birth cohorts										
1940–1955	2230	16.3	31	9.3	32	9.1	133	22.7	2426	16.2
1956–1970	4879	35.7	107	31.9	78	22.2	197	33.6	5261	35.2
1971–1985	4405	32.2	113	33.7	104	29.6	168	28.6	4790	32
1986–2001	2172	15.9	84	25.1	137	39	89	15.2	2482	16.6
Gender										
Male	6593	48.2	225	67.2	110	31.3	240	40.9	7168	47.9
Female	7093	51.8	110	32.8	241	68.7	347	59.1	7791	52.1
Education^a^										
Low	1392	10.2	27	8.1	42	12	98	16.7	1559	10.4
Medium	8151	59.6	175	52.2	204	58.1	386	65.8	8916	59.6
High	3727	27.2	129	38.5	97	27.6	76	13	4029	26.9
Still in education	63	0.5	2	0.6	1	0.3	4	0.7	70	0.5
No answer	353	2.6	2	0.6	7	2	23	3.9	385	2.6
Place of residence, age 15										
City	3235	23.6	81	24.2	117	33.3	104	17.7	3537	23.6
Other^b^	10,451	76.4	254	75.8	234	66.7	483	82.3	11,422	76.4
Socialised in the GDR^c^										
Yes	2557	18.7	21	6.3	36	10.3	100	17	2714	18.1
No, other	11,129	81.3	314	93.7	315	89.7	487	83	12,245	81.9
Father’s education										
Low	1312	9.6	23	6.9	27	7.7	97	16.5	1459	9.8
Medium	9400	68.7	227	67.8	220	62.7	374	63.7	10,221	68.3
High	2089	15.3	63	18.8	74	21.1	48	8.2	2274	15.2
Other^b^	885	6.5	22	6.6	30	8.6	68	11.6	1005	6.7
*N* and row %	13,686	91.5	335	2.2	351	2.3	587	3.9	14,959	100

Percentages may not sum to 100 due to rounding

Data source: German Socio-Economic Panel, survey years 2016 and 2019

*GDR* German Democratic Republic

^a^Education is used as a time-varying covariate; here: education at time of interview

^b^Including no answer, not asked, not applicable, don’t know (only father’s education)

3,

**Table 4:** Description of the sample used to analyse the process of first cohabitation

	Sexual orientation								All	
	Heterosexual		Homosexual		Bisexual		Other, no answer			
	*N*	Col. %	*N*	Col. %	*N*	Col. %	*N*	Col. %	*N*	Col. %
Birth cohorts										
1940–1955	2301	12.9	33	8.1	33	7	137	15.1	2504	12.7
1956–1970	5247	29.4	119	29.2	81	17.1	242	26.6	5689	28.9
1971–1985	5152	28.8	126	30.9	112	23.7	220	24.2	5610	28.5
1986–2001	5172	28.9	130	31.8	247	52.2	311	34.2	5860	29.8
Gender										
Male	8700	48.7	266	65.2	140	29.6	409	44.9	9515	48.4
Female	9172	51.3	142	34.8	333	70.4	501	55.1	10,148	51.6
Education^a^										
Low	2029	11.4	32	7.8	68	14.4	171	18.8	2300	11.7
Medium	10,059	56.3	205	50.3	260	54.9	514	56.5	11,038	56.1
High	4433	24.8	149	36.5	107	22.6	107	11.8	4796	24.4
Still in education	405	2.3	8	2	15	3.2	25	2.7	453	2.3
No answer	946	5.3	14	3.4	23	4.9	93	10.2	1076	5.5
Place of residence, age 15										
City	4119	23.1	99	24.3	145	30.7	150	16.5	4513	22.9
Other^b^	13,753	76.9	309	75.7	328	69.3	760	83.5	15,150	77.1
Socialised in the GDR^c^										
Yes	2745	15.4	25	6.1	39	8.3	116	12.7	2935	14.9
No, other	15,127	84.6	383	93.9	434	91.7	794	87.3	16,738	85.1
Father’s education										
Low	1811	10.1	30	7.4	35	7.4	158	17.4	2034	10.3
Medium	12,170	68.1	273	66.9	294	62.1	568	62.4	13,305	67.7
High	2764	15.5	80	19.6	103	21.8	95	10.4	3042	15.5
Other^b^	1127	6.3	25	6.1	41	8.7	89	9.8	1282	6.5
*N* and row %	17,872	90.9	408	2.1	473	2.4	910	4.6	19,663	100

Percentages may not sum to 100 due to rounding

Data source: German Socio-Economic Panel, survey years 2016 and 2019

*GDR* German Democratic Republic

^a^Education is used as a time-varying covariate; here: education at time of interview

^b^Including no answer, not asked, not applicable, don’t know (only father’s education)

4,

**Table 5:** Discrete-time logit models predicting first partnership formation (β coefficients): Model 2 from Table 1 with additional interactions

	Model 2a	Model 2b
	+ IA Birth cohorts * Sexual orientation	+ IA Gender * Sexual orientation
*t*	− 0.150***	− 0.149***
ln(*t*)	2.144***	2.145***
Sexual orientation (ref. heterosexual)		
Homosexual	− 1.547***	− 1.373**
Bisexual	0.175	0.510^+^
Other, no answer	− 0.351	− 0.225
Process time × Sexual orientation		
*t* × homosexual	0.043*	0.028
*t* × bisexual	0.054*	0.045*
*t* × other, no answer	0.026	0.026
ln(*t*) × homosexual	0.111	0.146
ln(*t*) × bisexual	− 0.563**	− 0.559**
ln(*t*) × other, no answer	− 0.111	− 0.127
Birth cohorts (ref. 1956–1970)		
1940–1955	0.086**	0.088***
1971–1985	0.339***	0.350***
1986–2001	0.880***	0.896***
Birth cohorts × Sexual orientation		
1940–1955 × homosexual	− 0.587*	
1940–1955 × bisexual	− 0.032	
1940–1955 × other, no answer	0.243^+^	
1971–1985 × homosexual	0.040	
1971–1985 × bisexual	0.381*	
1971–1985 × other, no answer	0.116	
1986–2001 × homosexual	0.273	
1986–2001 × bisexual	0.201	
1986–2001 × other, no answer	0.148	
Gender (ref. male)		
Female	0.476***	0.481***
Gender × Sexual orientation		
Female × homosexual		− 0.151
Female × bisexual		− 0.124
Female × other, no answer		0.025
Education (ref. low)		
Medium	0.062	0.062
High	0.096^+^	0.096^+^
Still in education	0.033	0.032
No answer	− 0.282**	− 0.289**
Place of residence, age 15 (ref. other^a^)		
City	− 0.093***	− 0.092***
Socialised in the GDR (ref. no, other)		
Yes	0.002	0.003
Father’s education (ref. low)		
Medium	− 0.006	− 0.003
High	− 0.107**	− 0.104*
Other^a^	− 0.103*	− 0.101*
Constant	− 5.433***	− 5.448***
Log likelihood	− 43,678	− 43,688
*N* (person-years)	148,879	148,879

Data source: German Socio-Economic Panel, survey years 2016 and 2019

*IA* Interaction, *GDR* German Democratic Republic

^+^*p* < 0.10, * *p* < 0.05, ** *p* < 0.01, *** *p* < 0.001

^a^Including no answer, not asked, not applicable, don’t know (only father’s education)

5 and

**Table 6:** Discrete-time logit models predicting first coresidential union formation (β coefficients): Model 2 from Table 2 with additional interactions

	Model 2a	Model 2b
	+ IA Birth cohorts * Sexual orientation	+ IA Gender * Sexual orientation
*t*	− 0.102***	− 0.102***
ln(*t*)	1.235***	1.232***
Sexual orientation (ref. heterosexual)		
Homosexual	− 0.744*	− 0.793*
Bisexual	0.002	0.272
Other, no answer	0.073	− 0.095
Process time × Sexual orientation		
*t* × homosexual	0.028	0.014
*t* × bisexual	0.001	− 0.008
*t* × other, no answer	0.014	0.018
ln(*t*) × homosexual	− 0.057	− 0.004
ln(*t*) × bisexual	− 0.237	− 0.208
ln(*t*) × other, no answer	− 0.211	− 0.195
Birth cohorts (ref. 1956–1970)		
1940–1955	0.170***	0.174***
1971–1985	0.211***	0.214***
1986–2001	0.179***	0.170***
Birth cohorts × Sexual orientation		
1940–1955 × homosexual	− 0.787***	
1940–1955 × bisexual	0.116	
1940–1955 × other, no answer	0.255*	
1971–1985 × homosexual	− 0.115	
1971–1985 × bisexual	0.454**	
1971–1985 × other, no answer	− 0.030	
1986– 2001 × homosexual	0.195	
1986–2001 × bisexual	0.240	
1986–2001 × other, no answer	− 0.379*	
Gender (ref. male)		
Female	0.363***	0.359***
Gender × Sexual orientation		
Female × homosexual		0.018
Female × bisexual		− 0.012
Female × other, no answer		0.130
Education (ref. low)		
Medium	− 0.423***	− 0.425***
High	0.031	0.031
Still in education	0.063	0.062
No answer	− 0.208**	− 0.212**
Place of residence, age 15 (ref. other^a^)		
City	− 0.051*	− 0.051*
Socialised in the GDR (ref. no, other)		
Yes	0.046*	0.046*
Father’s education (ref. low)		
Medium	− 0.026	− 0.024
High	− 0.095**	− 0.093*
Other^a^	− 0.214***	− 0.212***
Constant	− 4.026***	− 4.021***
Log likelihood	− 51,824	− 51,844
*N* (person-years)	207,916	207,916

Data source: German Socio-Economic Panel, survey years 2016 and 2019

*IA* Interaction, *GDR* German Democratic Republic

^+^*p* < 0.10, * *p* < 0.05, ** *p* < 0.01, *** *p* < 0.001

^a^Including no answer, not asked, not applicable, don’t know (only father’s education)

6

See Figs. 7, 8, 9 and 10

## Footnotes

1 We use the term *sexual orientation* without wanting to reduce the lifestyles described by this term to the sexual aspect. We are also aware that homosexuality, bisexuality and heterosexuality are socio-cultural constructs based on a binary concept of gender identity, and that the actual diversity of lifestyles is more complex.

2 In the preceding years—from 2001 to 2016—same-sex couples in Germany could only enter into a registered civil partnership, which was not legally equivalent to marriage.

3 Estimates of the prevalence of non-heterosexual orientations in Germany and comparable European countries vary considerably but are generally in the low single-digit percentage range (e.g. Aspinall, 2009, pp. 50–58; Hayes et al., 2012; Kroh et al., 2017, p. 338), so that even large samples contain only a small number of LGB people.

4 Until the reform of Section 175 of the West German Criminal Code (StGB) in 1969, such acts were a criminal offence. However, although the provisions of Section 175 were relaxed in 1969, it was not fully repealed until 1994. In the German Democratic Republic (GDR), on the other hand, homosexuals were prosecuted less than in West Germany (Section 175 of the GDR Criminal Code [StGB-DDR] was abolished in 1968), but homophobic attitudes were widespread in the population there as well (Gammerl, 2010).

5 More precisely, we use SOEP-Core v36eu, https://doi.org/10.5684/soep.core.v36eu.

6 According to the questionnaire, the partnership biography includes both the current relationship and past relationships, irrespective of whether or not the respondents married in these relationships. Past relationships are defined as committed relationships that lasted six months or longer (see Kantar Public, 2020, for the exact wording of the German-language questionnaire).

7 Therefore, the earliest partnership reported in the partnership biography does not necessarily have to be the first partnership in the life course. However, the resulting error is likely to be small, as the overwhelming majority of the individuals we studied (over 90%, regardless of sexual orientation) did not report more than two previous partnerships.

8 As people remember their first cohabitation better than their first partnership, more people (with valid responses) are included in the second part of our analysis. This illustrates the problematic nature of retrospectively collected data, namely that they are susceptible to memory gaps and errors (e.g. Groves et al., 2009). Nevertheless, this should be of secondary importance for our study because we are less interested in the exact dates of the beginning of the first partnership and the first cohabitation, but rather in the differences between lesbians, gays and heterosexuals in this regard. There is no reason to assume that these groups remember previous partnerships differently.

9 The nine respondents in Sample Q with “other” gender or “no answer” were excluded from our analyses.

10 The differences according to gender do not overlap with the differences according to sexual orientation, but rather exist independently of them. This can be seen from the overall model, which we have expanded to include interactions between gender and sexual orientation (Appendix Table 5, Model 2b). The transition rates show that all groups differ from one another, and that the gender effect is similar for homosexual and heterosexual people (Appendix Fig. 8).

11 As in the case of the transition to the first partnership (see footnote 10), the differences by gender and the differences by sexual orientation also exist independently of each other in the transition to first cohabitation (Appendix Table 6, Model 2b). Heterosexual women have the highest transition rates into first cohabitation, followed by heterosexual men. The transition rates of lesbian women are lower, and those of gay men are the lowest (Appendix Fig. 10).
